# Morpholinium perchlorate

**DOI:** 10.1107/S1600536807068134

**Published:** 2008-01-09

**Authors:** Mikhail S. Grigoriev, Konstantin E. German, Alesia Ya. Maruk

**Affiliations:** a A. N. Frumkin Institute of Physical Chemistry and Electrochemistry, Russian Academy of Sciences, 31 Leninsky prospekt, 119991 Moscow, Russian Federation

## Abstract

In the title salt, C_4_H_10_NO^+^·ClO_4_^−^, which has three independent formula units, the cations are linked into chains along [100] by N—H⋯O hydrogen bonds. Each cation acts both as a donor and as an acceptor, and every cation makes one N—H⋯O hydrogen bond with a ClO_4_^−^ anion. The crystal studied was an inversion twin.

## Related literature

See Grigoriev *et al.* (2007 ▶) for the structure of morpholinium tetra­oxidorhenate(VII).
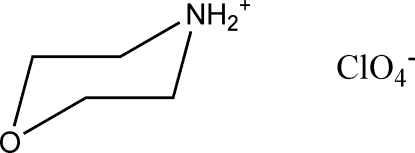


## Experimental

### 

#### Crystal data

C_4_H_10_NO^+^·ClO_4_^−^
*M* *_r_* = 187.58
Orthorhombic, 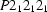

*a* = 8.1515 (3) Å
*b* = 9.5435 (4) Å
*c* = 28.9022 (12) Å
*V* = 2248.41 (16) Å^3^
*Z* = 12
Mo *K*α radiation
μ = 0.49 mm^−1^
*T* = 100 (2) K
0.24 × 0.20 × 0.16 mm

#### Data collection

Bruker Kappa APEXII area-detector diffractometer
Absorption correction: none
31159 measured reflections
6453 independent reflections
5928 reflections with *I* > 2σ(*I*)
*R*_int_ = 0.038

#### Refinement

*R*[*F* ^2^ > 2σ(*F* ^2^)] = 0.025
*wR*(*F* ^2^) = 0.062
*S* = 1.03
6453 reflections
323 parameters
6 restraints
H atoms treated by a mixture of independent and constrained refinement
Δρ_max_ = 0.30 e Å^−3^
Δρ_min_ = −0.32 e Å^−3^
Absolute structure: Flack (1983 ▶), 2793 Friedel pairs
Flack parameter: 0.42 (3)

### 

Data collection: *APEX2* (Bruker, 2006 ▶); cell refinement: *SAINT-Plus* (Bruker, 1998 ▶); data reduction: *SAINT-Plus*; program(s) used to solve structure: *SHELXS97* (Sheldrick, 1997*a* ▶); program(s) used to refine structure: *SHELXL97* (Sheldrick, 1997*a* ▶); molecular graphics: *SHELXTL* (Sheldrick, 1997*b* ▶); software used to prepare material for publication: *SHELXTL*.

## Supplementary Material

Crystal structure: contains datablocks global, I. DOI: 10.1107/S1600536807068134/ng2409sup1.cif

Structure factors: contains datablocks I. DOI: 10.1107/S1600536807068134/ng2409Isup2.hkl

Additional supplementary materials:  crystallographic information; 3D view; checkCIF report

## Figures and Tables

**Table 1:** Hydrogen-bond geometry (Å, °)

*D*—H⋯*A*	*D*—H	H⋯*A*	*D*⋯*A*	*D*—H⋯*A*
N1—H1*C*⋯O2	0.877 (9)	2.051 (13)	2.7872 (16)	141.0 (15)
N1—H1*D*⋯O15^i^	0.871 (9)	2.015 (10)	2.8642 (15)	164.7 (16)
N2—H2*C*⋯O14^ii^	0.884 (9)	1.980 (10)	2.8441 (14)	165.5 (16)
N2—H2*D*⋯O8	0.889 (9)	2.020 (11)	2.8465 (15)	154.1 (15)
N3—H3*C*⋯O13^i^	0.878 (9)	2.004 (10)	2.8690 (15)	168.0 (17)
N3—H3*D*⋯O9	0.870 (9)	2.039 (13)	2.7895 (17)	143.9 (16)

Symmetry codes: (i) 
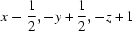
; (ii) 
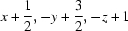
.

**Fig. 1.**
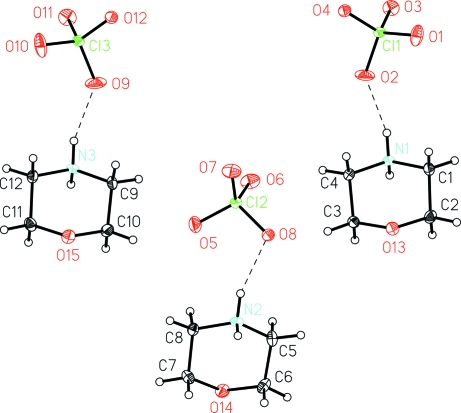
A view of (I), showing the atom-labelling scheme. Displacement ellipsoids are drawn at the 50% probability level and H atoms are represented by circles of arbitrary size. Dashed lines indicate the hydrogen-bonding interaction.

**Fig. 2.**
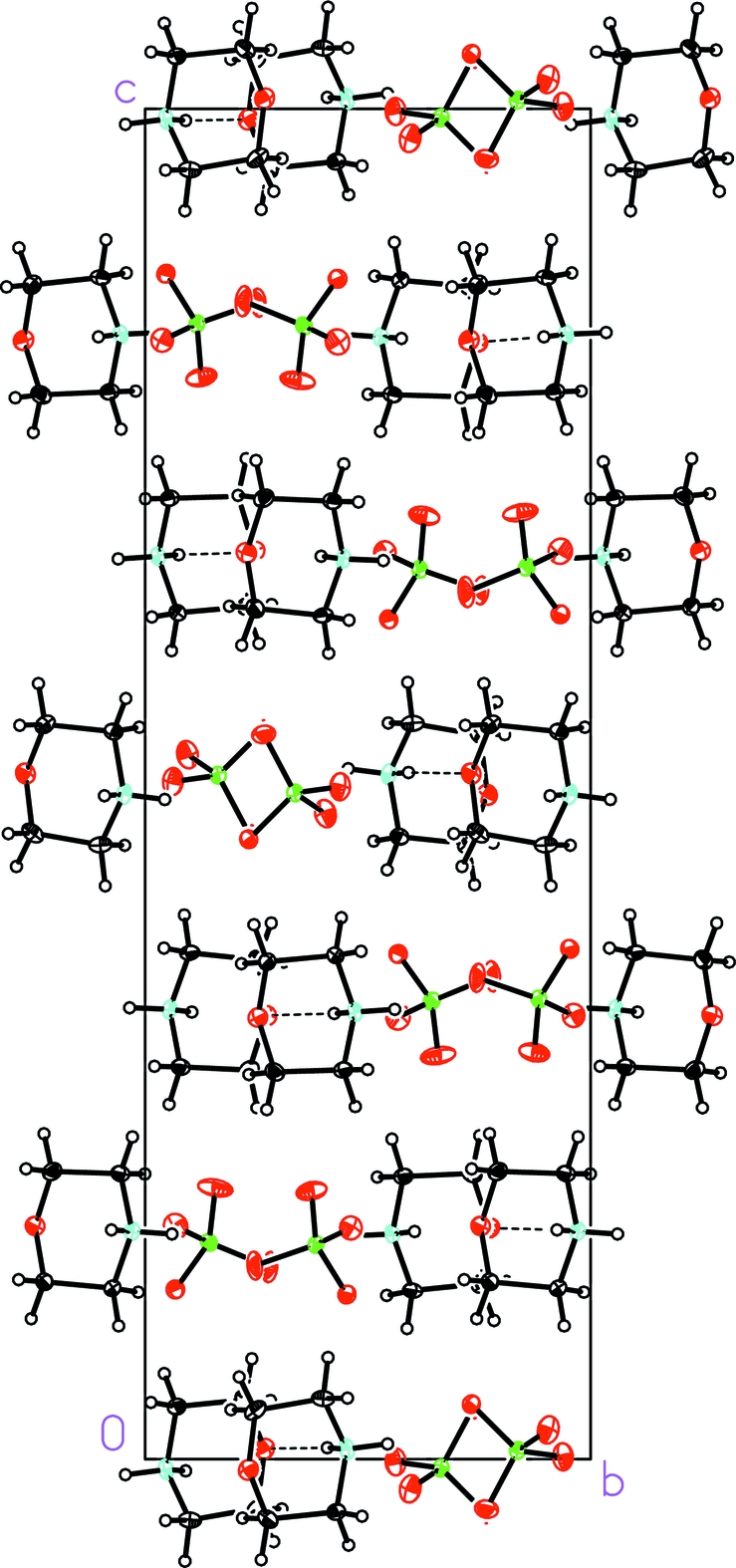
The unit cell of (I).

**Fig. 3.**
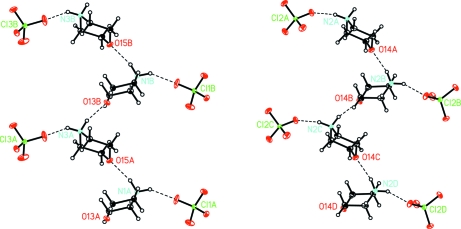
The comparison of two different types of chains.

## supplementary crystallographic information

### Comment

The title compound, (I) (Fig. 1), contains slightly distorted tetrahedral ClO_4_^-^ anions with Cl—O distances from 1.4353 (11) to 1.4496 (10) Å. Morpholinium cations have chair conformation.

The structure of (I) can be described as alternating cationic and anionic layers parallel to the (013) plane (Fig. 2).

Every morpholinium cation acts as proton donor in two hydrogen bonds, acceptors being O atoms of another morpholinium cation and ClO_4_^-^ anion (Fig. 3, Table 1).

Morpholinium tetraoxidochlorate(VII) contains three independent formula units, which make two different types of zigzag chains in the [100] direction by N—H···O hydrogen bonds between cations (Fig. 3). The first type is formed by one formula unit and the second type is formed by two remaining formula units. As it is nseen in Fig. 3, two types of chains have different orientation of ClO_4_^-^ anions.

The structure of morpholinium tetraoxidorhenate(VII) (Grigoriev *et al.*, 2007) also contains alternating cationic and anionic layers with hydrogen bonds in perpendicular direction. But in contrast to (I), O atoms of morpholinium in (C_4_H_10_NO)[ReO_4_] do not participate in hydrogen-bonding.

### Experimental

Synthesis of (I) was carried out as a neutralization reaction by dissolution of stoichiometric quantity of morpholine under intensive stirring in 0.92 *M* water solution of HClO_4_ at room temperature, followed by evaporation of the resulting solution at temperature 323 K.

### Refinement

The H atoms of CH_2_ groups were refined in idealized geometrical positions with displacement parameters being equal to 1.2 times *U*_eq_ of the attached C atoms. The H atoms of NH_2_ were located on difference Fourier map and refined with displacement parameters being equal to 1.2 times *U*_eq_ of the attached N atoms and N—H distances restrained to 0.88 (1) Å.

The Flack parameter was explicitly refined. The absolute structure was selected on the basis of the lower Flack parameter; the structure is a racemic twin.

### Crystal data

**Table:**

C_4_H_10_NO^+^·ClO_4_^–^	*F*_000_ = 1176
*M**_r_* = 187.58	*D*_x_ = 1.662 Mg m^−^^3^
Orthorhombic, *P*2_1_2_1_2_1_	Mo *K*α radiation λ = 0.71073 Å
Hall symbol: P 2ac 2ab	Cell parameters from 7054 reflections
*a* = 8.1515 (3) Å	θ = 2.3–30.0º
*b* = 9.5435 (4) Å	µ = 0.49 mm^−^^1^
*c* = 28.9022 (12) Å	*T* = 100 (2) K
*V* = 2248.41 (16) Å^3^	Fragment, colourless
*Z* = 12	0.24 × 0.20 × 0.16 mm

### Data collection

**Table:**

Bruker KappaAPEXII area-detector diffractometer	5928 reflections with *I* > 2σ(*I*)
Radiation source: fine-focus sealed tube	*R*_int_ = 0.038
Monochromator: graphite	θ_max_ = 30.0º
*T* = 100(2) K	θ_min_ = 2.3º
ω and φ scans	*h* = −11→11
Absorption correction: none (SADABS; Sheldrick, 2004?)	*k* = −13→13
31159 measured reflections	*l* = −40→40
6453 independent reflections	

### Refinement

**Table:**

Refinement on *F*^2^	Hydrogen site location: mixed
Least-squares matrix: full	H atoms treated by a mixture of independent and constrained refinement
*R*[*F*^2^ > 2σ(*F*^2^)] = 0.025	*w* = 1/[σ^2^(*F*_o_^2^) + (0.0368*P*)^2^ + 0.1234*P*] where *P* = (*F*_o_^2^ + 2*F*_c_^2^)/3
*wR*(*F*^2^) = 0.062	(Δ/σ)_max_ = 0.001
*S* = 1.03	Δρ_max_ = 0.30 e Å^−^^3^
6453 reflections	Δρ_min_ = −0.32 e Å^−^^3^
323 parameters	Extinction correction: none
6 restraints	Absolute structure: Flack (1983), 2793 Friedel pairs
Primary atom site location: structure-invariant direct methods	Flack parameter: 0.42 (3)
Secondary atom site location: difference Fourier map	

### Special details

**Table:**

Geometry. All e.s.d.'s (except the e.s.d. in the dihedral angle between two l.s. planes) are estimated using the full covariance matrix. The cell e.s.d.'s are taken into account individually in the estimation of e.s.d.'s in distances, angles and torsion angles; correlations between e.s.d.'s in cell parameters are only used when they are defined by crystal symmetry. An approximate (isotropic) treatment of cell e.s.d.'s is used for estimating e.s.d.'s involving l.s. planes.
Refinement. Refinement of *F*^2^ against ALL reflections. The weighted *R*-factor *wR* and goodness of fit *S* are based on *F*^2^, conventional *R*-factors *R* are based on *F*, with *F* set to zero for negative *F*^2^. The threshold expression of *F*^2^ > σ(*F*^2^) is used only for calculating *R*-factors(gt) *etc*. and is not relevant to the choice of reflections for refinement. *R*-factors based on *F*^2^ are statistically about twice as large as those based on *F*, and *R*- factors based on ALL data will be even larger.

### Fractional atomic coordinates and isotropic or equivalent isotropic displacement parameters (Å^2^)

**Table:**

	*x*	*y*	*z*	*U*_iso_*/*U*_eq_	
Cl1	0.57617 (4)	−0.38371 (3)	0.659062 (11)	0.01229 (6)	
Cl2	0.37184 (4)	0.16395 (3)	0.506534 (10)	0.01225 (6)	
Cl3	0.57257 (4)	−0.35661 (3)	0.339378 (11)	0.01296 (7)	
O1	0.68287 (14)	−0.37350 (14)	0.69869 (4)	0.0261 (3)	
O2	0.53026 (15)	−0.24599 (12)	0.64345 (5)	0.0297 (3)	
O3	0.43207 (14)	−0.46173 (11)	0.67131 (4)	0.0207 (2)	
O4	0.66220 (12)	−0.45314 (11)	0.62201 (3)	0.0157 (2)	
O5	0.34519 (14)	0.23379 (12)	0.46318 (4)	0.0237 (2)	
O6	0.22474 (13)	0.09516 (12)	0.52183 (4)	0.0246 (2)	
O7	0.50117 (12)	0.06236 (11)	0.50191 (4)	0.0248 (2)	
O8	0.41766 (13)	0.26762 (11)	0.54084 (4)	0.0189 (2)	
O9	0.53099 (16)	−0.22094 (13)	0.35767 (5)	0.0342 (3)	
O10	0.67421 (14)	−0.34228 (16)	0.29901 (4)	0.0321 (3)	
O11	0.42568 (14)	−0.43179 (12)	0.32752 (4)	0.0236 (2)	
O12	0.66235 (13)	−0.43202 (11)	0.37422 (3)	0.0168 (2)	
O13	0.69481 (11)	0.24689 (11)	0.67280 (4)	0.0155 (2)	
O14	0.22067 (11)	0.76837 (11)	0.49220 (3)	0.0171 (2)	
O15	0.69469 (12)	0.27281 (11)	0.32867 (4)	0.0165 (2)	
N1	0.45845 (14)	0.02905 (12)	0.66878 (4)	0.0119 (2)	
H1C	0.434 (2)	−0.0603 (10)	0.6668 (6)	0.014*	
H1D	0.3666 (14)	0.0755 (16)	0.6699 (6)	0.014*	
N2	0.44656 (13)	0.54724 (12)	0.50755 (4)	0.0135 (2)	
H2C	0.5419 (14)	0.5912 (16)	0.5086 (6)	0.016*	
H2D	0.462 (2)	0.4558 (10)	0.5114 (6)	0.016*	
N3	0.45865 (14)	0.05497 (12)	0.33319 (4)	0.0122 (2)	
H3C	0.3684 (15)	0.1049 (17)	0.3313 (6)	0.015*	
H3D	0.440 (2)	−0.0347 (10)	0.3344 (6)	0.015*	
C1	0.55582 (18)	0.05548 (15)	0.71163 (5)	0.0144 (3)	
H1A	0.492 (2)	0.0284 (19)	0.7388 (6)	0.017*	
H1B	0.646 (2)	0.0006 (19)	0.7113 (6)	0.017*	
C2	0.60347 (17)	0.20873 (15)	0.71312 (5)	0.0164 (3)	
H2A	0.5033	0.2671	0.7151	0.020*	
H2B	0.6705	0.2268	0.7411	0.020*	
C3	0.59875 (17)	0.22590 (15)	0.63150 (5)	0.0143 (3)	
H3A	0.6628	0.2556	0.6041	0.017*	
H3B	0.4981	0.2838	0.6330	0.017*	
C4	0.55258 (18)	0.07310 (14)	0.62683 (5)	0.0144 (3)	
H4A	0.4847	0.0594	0.5988	0.017*	
H4B	0.6528	0.0153	0.6237	0.017*	
C5	0.33638 (17)	0.60033 (17)	0.54496 (5)	0.0167 (3)	
H5A	0.3905	0.5894	0.5754	0.020*	
H5B	0.2331	0.5458	0.5453	0.020*	
C6	0.29959 (18)	0.75241 (16)	0.53623 (5)	0.0188 (3)	
H6A	0.2273	0.7889	0.5610	0.023*	
H6B	0.4028	0.8070	0.5367	0.023*	
C7	0.32779 (17)	0.72437 (16)	0.45577 (5)	0.0164 (3)	
H7A	0.4293	0.7813	0.4564	0.020*	
H7B	0.2736	0.7395	0.4255	0.020*	
C8	0.37108 (18)	0.57105 (15)	0.46105 (4)	0.0148 (3)	
H8A	0.2710	0.5131	0.4578	0.018*	
H8B	0.4492	0.5431	0.4365	0.018*	
C9	0.55216 (18)	0.10032 (15)	0.37512 (4)	0.0150 (3)	
H9A	0.6521	0.0423	0.3786	0.018*	
H9B	0.4837	0.0879	0.4031	0.018*	
C10	0.59916 (17)	0.25284 (15)	0.36984 (5)	0.0162 (3)	
H10A	0.4988	0.3110	0.3682	0.019*	
H10B	0.6634	0.2832	0.3971	0.019*	
C11	0.60345 (17)	0.23347 (15)	0.28832 (5)	0.0168 (3)	
H11A	0.6704	0.2510	0.2603	0.020*	
H11B	0.5029	0.2913	0.2862	0.020*	
C12	0.55698 (18)	0.07970 (14)	0.29050 (5)	0.0146 (3)	
H12A	0.4920	0.0539	0.2629	0.018*	
H12B	0.6572	0.0211	0.2911	0.018*	

### Atomic displacement parameters (Å^2^)

**Table:**

	*U*^11^	*U*^22^	*U*^33^	*U*^12^	*U*^13^	*U*^23^
Cl1	0.01222 (14)	0.01077 (13)	0.01386 (13)	−0.00072 (11)	0.00049 (11)	−0.00074 (11)
Cl2	0.01095 (11)	0.01167 (13)	0.01412 (13)	0.00027 (10)	−0.00132 (11)	−0.00073 (11)
Cl3	0.01248 (14)	0.01263 (14)	0.01377 (13)	−0.00160 (11)	−0.00006 (11)	0.00151 (11)
O1	0.0178 (5)	0.0441 (8)	0.0165 (5)	−0.0076 (5)	−0.0015 (4)	−0.0063 (5)
O2	0.0395 (7)	0.0108 (5)	0.0389 (7)	0.0094 (5)	0.0117 (5)	0.0024 (5)
O3	0.0130 (4)	0.0230 (5)	0.0262 (5)	−0.0054 (4)	0.0027 (4)	0.0000 (4)
O4	0.0170 (5)	0.0146 (5)	0.0155 (4)	0.0036 (4)	0.0009 (4)	−0.0010 (4)
O5	0.0302 (6)	0.0260 (6)	0.0150 (5)	0.0064 (5)	−0.0028 (4)	0.0020 (4)
O6	0.0154 (5)	0.0216 (6)	0.0368 (6)	−0.0073 (4)	0.0043 (4)	−0.0036 (5)
O7	0.0189 (5)	0.0193 (5)	0.0361 (6)	0.0093 (4)	0.0022 (5)	0.0007 (5)
O8	0.0271 (5)	0.0143 (5)	0.0153 (5)	−0.0058 (4)	−0.0048 (4)	−0.0006 (4)
O9	0.0453 (8)	0.0124 (5)	0.0449 (8)	0.0121 (5)	−0.0133 (6)	−0.0033 (5)
O10	0.0233 (6)	0.0579 (9)	0.0150 (5)	−0.0133 (6)	0.0023 (4)	0.0081 (6)
O11	0.0126 (5)	0.0272 (6)	0.0311 (6)	−0.0067 (4)	−0.0034 (4)	0.0013 (5)
O12	0.0195 (5)	0.0152 (5)	0.0158 (4)	0.0042 (4)	−0.0018 (4)	0.0001 (4)
O13	0.0128 (4)	0.0172 (5)	0.0166 (5)	−0.0049 (4)	−0.0022 (4)	0.0006 (4)
O14	0.0143 (4)	0.0174 (5)	0.0196 (5)	0.0057 (4)	−0.0010 (4)	−0.0015 (4)
O15	0.0129 (5)	0.0185 (5)	0.0181 (5)	−0.0061 (4)	0.0007 (4)	−0.0014 (4)
N1	0.0104 (5)	0.0102 (5)	0.0152 (5)	0.0001 (4)	−0.0003 (4)	−0.0012 (4)
N2	0.0108 (5)	0.0101 (5)	0.0195 (6)	0.0003 (4)	−0.0001 (4)	0.0018 (4)
N3	0.0115 (6)	0.0092 (5)	0.0159 (5)	0.0000 (4)	0.0008 (4)	0.0014 (4)
C1	0.0158 (7)	0.0162 (7)	0.0112 (6)	−0.0004 (5)	−0.0004 (5)	0.0012 (5)
C2	0.0180 (7)	0.0167 (7)	0.0145 (6)	−0.0036 (5)	−0.0015 (5)	−0.0027 (5)
C3	0.0163 (7)	0.0131 (6)	0.0133 (6)	−0.0010 (5)	−0.0013 (5)	0.0009 (5)
C4	0.0187 (7)	0.0127 (6)	0.0118 (6)	0.0001 (5)	−0.0002 (5)	−0.0013 (5)
C5	0.0146 (6)	0.0244 (7)	0.0109 (5)	−0.0002 (5)	0.0002 (5)	0.0009 (5)
C6	0.0183 (7)	0.0210 (8)	0.0170 (7)	0.0030 (5)	−0.0020 (5)	−0.0072 (6)
C7	0.0159 (6)	0.0181 (7)	0.0152 (6)	0.0024 (5)	0.0017 (5)	0.0045 (5)
C8	0.0166 (6)	0.0156 (7)	0.0123 (6)	0.0012 (5)	0.0026 (5)	−0.0010 (5)
C9	0.0174 (7)	0.0180 (7)	0.0097 (5)	0.0004 (5)	0.0004 (5)	0.0014 (5)
C10	0.0169 (7)	0.0156 (7)	0.0161 (6)	−0.0014 (5)	0.0009 (5)	−0.0024 (5)
C11	0.0169 (7)	0.0185 (7)	0.0150 (6)	−0.0038 (5)	0.0000 (5)	0.0031 (5)
C12	0.0156 (6)	0.0167 (7)	0.0116 (6)	−0.0013 (5)	0.0011 (5)	−0.0009 (5)

### Geometric parameters (Å, °)

**Table:**

Cl1—O3	1.4351 (11)	C1—C2	1.514 (2)
Cl1—O2	1.4391 (12)	C1—H1A	0.975 (18)
Cl1—O4	1.4415 (10)	C1—H1B	0.900 (18)
Cl1—O1	1.4415 (11)	C2—H2A	0.9900
Cl2—O5	1.4359 (11)	C2—H2B	0.9900
Cl2—O6	1.4367 (11)	C3—C4	1.5121 (19)
Cl2—O7	1.4385 (10)	C3—H3A	0.9900
Cl2—O8	1.4496 (10)	C3—H3B	0.9900
Cl3—O11	1.4374 (11)	C4—H4A	0.9900
Cl3—O10	1.4376 (11)	C4—H4B	0.9900
Cl3—O12	1.4379 (10)	C5—C6	1.503 (2)
Cl3—O9	1.4390 (13)	C5—H5A	0.9900
O13—C2	1.4301 (17)	C5—H5B	0.9900
O13—C3	1.4415 (16)	C6—H6A	0.9900
O14—C7	1.4308 (16)	C6—H6B	0.9900
O14—C6	1.4342 (17)	C7—C8	1.513 (2)
O15—C11	1.4333 (17)	C7—H7A	0.9900
O15—C10	1.4349 (17)	C7—H7B	0.9900
N1—C1	1.4926 (17)	C8—H8A	0.9900
N1—C4	1.4951 (17)	C8—H8B	0.9900
N1—H1C	0.877 (9)	C9—C10	1.513 (2)
N1—H1D	0.871 (9)	C9—H9A	0.9900
N2—C5	1.4943 (18)	C9—H9B	0.9900
N2—C8	1.4955 (17)	C10—H10A	0.9900
N2—H2C	0.884 (9)	C10—H10B	0.9900
N2—H2D	0.889 (9)	C11—C12	1.5169 (19)
N3—C12	1.4903 (17)	C11—H11A	0.9900
N3—C9	1.4956 (18)	C11—H11B	0.9900
N3—H3C	0.878 (9)	C12—H12A	0.9900
N3—H3D	0.870 (9)	C12—H12B	0.9900
			
O3—Cl1—O2	109.78 (7)	H3A—C3—H3B	108.2
O3—Cl1—O4	110.06 (6)	N1—C4—C3	109.05 (11)
O2—Cl1—O4	108.26 (7)	N1—C4—H4A	109.9
O3—Cl1—O1	109.45 (7)	C3—C4—H4A	109.9
O2—Cl1—O1	110.14 (8)	N1—C4—H4B	109.9
O4—Cl1—O1	109.14 (7)	C3—C4—H4B	109.9
O5—Cl2—O6	110.75 (7)	H4A—C4—H4B	108.3
O5—Cl2—O7	110.04 (7)	N2—C5—C6	109.01 (12)
O6—Cl2—O7	109.41 (7)	N2—C5—H5A	109.9
O5—Cl2—O8	108.60 (7)	C6—C5—H5A	109.9
O6—Cl2—O8	108.45 (7)	N2—C5—H5B	109.9
O7—Cl2—O8	109.55 (7)	C6—C5—H5B	109.9
O11—Cl3—O10	109.51 (7)	H5A—C5—H5B	108.3
O11—Cl3—O12	109.94 (7)	O14—C6—C5	109.94 (12)
O10—Cl3—O12	108.83 (7)	O14—C6—H6A	109.7
O11—Cl3—O9	109.91 (8)	C5—C6—H6A	109.7
O10—Cl3—O9	110.39 (9)	O14—C6—H6B	109.7
O12—Cl3—O9	108.24 (7)	C5—C6—H6B	109.7
C2—O13—C3	110.89 (10)	H6A—C6—H6B	108.2
C7—O14—C6	110.37 (10)	O14—C7—C8	110.61 (11)
C11—O15—C10	111.00 (10)	O14—C7—H7A	109.5
C1—N1—C4	110.64 (10)	C8—C7—H7A	109.5
C1—N1—H1C	109.7 (11)	O14—C7—H7B	109.5
C4—N1—H1C	109.6 (11)	C8—C7—H7B	109.5
C1—N1—H1D	109.9 (11)	H7A—C7—H7B	108.1
C4—N1—H1D	109.2 (11)	N2—C8—C7	109.49 (11)
H1C—N1—H1D	107.8 (16)	N2—C8—H8A	109.8
C5—N2—C8	110.58 (10)	C7—C8—H8A	109.8
C5—N2—H2C	110.1 (11)	N2—C8—H8B	109.8
C8—N2—H2C	108.7 (11)	C7—C8—H8B	109.8
C5—N2—H2D	109.2 (11)	H8A—C8—H8B	108.2
C8—N2—H2D	108.7 (11)	N3—C9—C10	109.02 (11)
H2C—N2—H2D	109.6 (15)	N3—C9—H9A	109.9
C12—N3—C9	110.54 (11)	C10—C9—H9A	109.9
C12—N3—H3C	108.1 (11)	N3—C9—H9B	109.9
C9—N3—H3C	108.8 (11)	C10—C9—H9B	109.9
C12—N3—H3D	106.5 (11)	H9A—C9—H9B	108.3
C9—N3—H3D	109.9 (12)	O15—C10—C9	110.41 (11)
H3C—N3—H3D	113.0 (17)	O15—C10—H10A	109.6
N1—C1—C2	108.86 (11)	C9—C10—H10A	109.6
N1—C1—H1A	109.9 (10)	O15—C10—H10B	109.6
C2—C1—H1A	111.7 (11)	C9—C10—H10B	109.6
N1—C1—H1B	109.0 (11)	H10A—C10—H10B	108.1
C2—C1—H1B	110.8 (12)	O15—C11—C12	110.44 (12)
H1A—C1—H1B	106.5 (15)	O15—C11—H11A	109.6
O13—C2—C1	110.88 (11)	C12—C11—H11A	109.6
O13—C2—H2A	109.5	O15—C11—H11B	109.6
C1—C2—H2A	109.5	C12—C11—H11B	109.6
O13—C2—H2B	109.5	H11A—C11—H11B	108.1
C1—C2—H2B	109.5	N3—C12—C11	108.78 (11)
H2A—C2—H2B	108.1	N3—C12—H12A	109.9
O13—C3—C4	110.07 (11)	C11—C12—H12A	109.9
O13—C3—H3A	109.6	N3—C12—H12B	109.9
C4—C3—H3A	109.6	C11—C12—H12B	109.9
O13—C3—H3B	109.6	H12A—C12—H12B	108.3
C4—C3—H3B	109.6		
			
C4—N1—C1—C2	56.08 (15)	C6—O14—C7—C8	61.45 (14)
C3—O13—C2—C1	60.92 (15)	C5—N2—C8—C7	54.51 (14)
N1—C1—C2—O13	−57.76 (15)	O14—C7—C8—N2	−56.81 (14)
C2—O13—C3—C4	−61.05 (15)	C12—N3—C9—C10	56.67 (14)
C1—N1—C4—C3	−56.79 (15)	C11—O15—C10—C9	60.83 (14)
O13—C3—C4—N1	58.36 (14)	N3—C9—C10—O15	−57.92 (14)
C8—N2—C5—C6	−56.06 (14)	C10—O15—C11—C12	−61.04 (14)
C7—O14—C6—C5	−62.99 (15)	C9—N3—C12—C11	−56.71 (15)
N2—C5—C6—O14	59.76 (15)	O15—C11—C12—N3	58.34 (15)

### Hydrogen-bond geometry (Å, °)

**Table:**

*D*—H···*A*	*D*—H	H···*A*	*D*···*A*	*D*—H···*A*
N1—H1C···O2	0.877 (9)	2.051 (13)	2.7872 (16)	141.0 (15)
N1—H1D···O15^i^	0.871 (9)	2.015 (10)	2.8642 (15)	164.7 (16)
N2—H2C···O14^ii^	0.884 (9)	1.980 (10)	2.8441 (14)	165.5 (16)
N2—H2D···O8	0.889 (9)	2.020 (11)	2.8465 (15)	154.1 (15)
N3—H3C···O13^i^	0.878 (9)	2.004 (10)	2.8690 (15)	168.0 (17)
N3—H3D···O9	0.870 (9)	2.039 (13)	2.7895 (17)	143.9 (16)

Symmetry codes: (i) *x*−1/2, −*y*+1/2, −*z*+1; (ii) *x*+1/2, −*y*+3/2, −*z*+1.
